# The Burden of Inflammatory Bowel Disease in Gulf Cooperation Council Countries from 1990 to 2021 with Forecasting Projections to 2030: A Global Burden of Disease Study

**DOI:** 10.3390/healthcare13233104

**Published:** 2025-11-28

**Authors:** Ahmed K. Alsaif, Jawad S. Alnajjar, Faisal A. Al-Harbi, Ahmed A. Alsirhani, Sultan S. Alruqaie, Abdulaziz T. Alturki, Mohammed A. Basuhail, Abdulrhman K. Alabdulqader, Ahmed A. Albadrani, Ahmed Y. Azzam

**Affiliations:** 1 College of Medicine, Al-Rayan National Colleges, Al-Madinah 42541, Saudi Arabia; a.k.alsaif.md@gmail.com; 2 College of Medicine, King Faisal University, Al-Ahsa 31982, Saudi Arabia; jawd.alnajjar@gmail.com; 3 College of Medicine, Qassim University, Buraydah 52571, Saudi Arabia; dr.sultan2025@gmail.com (S.S.A.); 1abdulazizalturki1@gmail.com (A.T.A.); 4 Department of Dermatology, Antonius Ziekenhuis Sneek, 8601 ZK Sneek, The Netherlands; alsir7ani@gmail.com; 5 Faculty of Medical Sciences, University of Groningen, 9712 CP Groningen, The Netherlands; 6 Department of Internal Medicine, College of Medicine, King Faisal University, Al-Ahsa 31982, Saudi Arabia; alabdulqader@kfu.edu.sa; 7 Department of Internal Medicine, College of Medicine, Prince Sattam bin Abdulaziz University, Al-Kharj 16278, Saudi Arabia; a.albadrani@psau.edu.sa; 8 ASIDE Healthcare, Lewes, DE 19958, USA; ahmedyazzam@gmail.com; 9 Division of Global Health and Public Health, School of Nursing, Midwifery and Public Health, University of Suffolk, Ipswich IP4 1QJ, UK

**Keywords:** inflammatory bowel disease, global burden of disease, epidemiology, Gulf Cooperation Council, healthcare modeling

## Abstract

**Background:** Inflammatory bowel disease (IBD) represents a growing health challenge in regions undergoing socioeconomic transition. This study characterized IBD epidemiology across Gulf Cooperation Council (GCC) countries, forecasted future burden, and identified country-specific determinants to inform targeted health policy interventions. **Methods:** IBD data from the Global Burden of Disease study (1990–2021) were analyzed for all six GCC countries using descriptive epidemiology, temporal trend analysis, three forecasting models (Age-Period-Cohort, Joinpoint regression, Bayesian age-standardized rate modeling), and regression analyses to identify socioeconomic, environmental, gender-specific, and healthcare system factors associated with IBD burden variation. **Results:** Age-standardized IBD prevalence rates varied across GCC countries (28.92–42.93 per 100,000), with Qatar and the United Arab Emirates (UAE) showing the highest rates and fastest increases (967% and 898% since 1990). Kuwait uniquely demonstrated stable or slightly declining trends. Significant female predominance emerged in most countries (male ratio 0.70–0.91) with strong birth cohort effects (1970s cohorts showing 18–48% higher risk than 1950s cohorts). IBD manifested primarily as disability rather than a mortality burden. Projections indicate continued increases through 2030, potentially reaching 55–59 per 100,000 in Qatar and the UAE. Fast food outlet density, processed food imports, antibiotic consumption, and female vitamin D deficiency were the most significant modifiable risk factors. **Conclusions:** The GCC region faces a growing IBD epidemiological profile characterized by rising burden, female predominance, and generational differences in risk observations. Identification of modifiable determinants provides actionable targets for intervention, while country-specific projections offer a foundation for healthcare planning to address this challenge.

## 1. Introduction

Inflammatory bowel disease (IBD), comprising primarily Crohn’s disease and ulcerative colitis, represents a significant global health challenge characterized by chronic gastrointestinal inflammation with relapsing-remitting patterns [1]. IBD prevalence has risen across multiple countries in recent decades, with historically the highest rates in Western industrialized nations [2]. However, an epidemiological transition is occurring in previously low-incidence regions, including the Middle East [3]. The Gulf Cooperation Council (GCC) countries, Saudi Arabia, United Arab Emirates (UAE), Kuwait, Qatar, Oman, and Bahrain, have undergone rapid socioeconomic development, urbanization, and lifestyle westernization in recent decades, creating environmental conditions potentially conducive to IBD emergence [3].

Despite abundant epidemiological data from North America and Europe, comprehensive evidence on IBD burden, trends, and projections remains limited in the GCC region [4]. The unique demographic composition, rapid lifestyle transitions, and evolving healthcare systems of GCC countries necessitate region-specific investigations to inform appropriate public health responses [5]. IBD typically affects individuals during their most productive years and requires lifelong management, underscoring the importance of evidence-based projections for resource allocation and healthcare planning. Identifying modifiable risk factors specific to the GCC population could enable targeted prevention strategies in this increasingly affected region.

This study addresses these gaps through a comprehensive analysis of IBD epidemiology across all six GCC countries using the Global Burden of Disease (GBD) framework. Study objectives include (1) characterizing baseline demographics and current IBD burden with cross-country and gender comparisons; (2) analyzing temporal trends from 1990 to 2021 to identify inflection points and patterns of change across three decades of regional development; (3) forecasting IBD burden through 2030 using multiple modeling approaches to inform healthcare planning and resource allocation; and (4) investigating socioeconomic, environmental, gender-specific, and healthcare system factors associated with IBD burden variation within the GCC region [6].

## 2. Methods

### 2.1. Data Sources and Study Design

**Figure 1:**
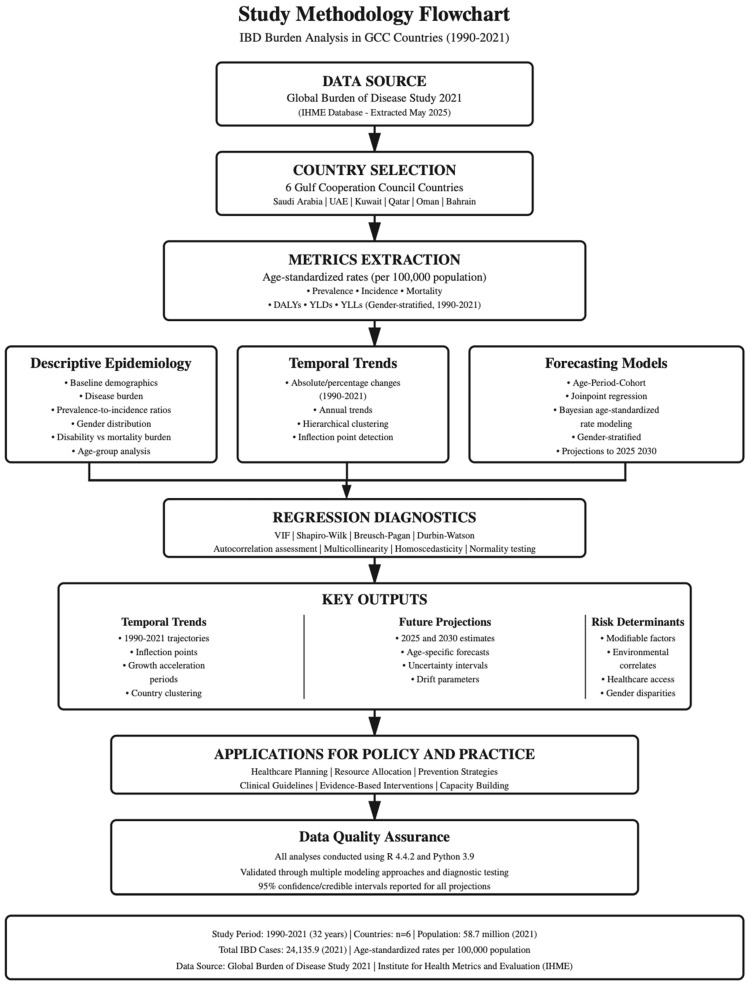
Flowchart of study process pipeline.

IBD data for GCC countries were extracted from the GBD 2021 study database in May 2025 via the Institute for Health Metrics and Evaluation (IHME) visualization hub (Figure 1) [7]. This study utilized publicly available, aggregated data from GBD 2021, which is freely accessible for download via the IHME visualization hub for research purposes. Data were extracted via https://vizhub.healthdata.org/gbd-results/ (accessed on 1 May 2025). This study utilized publicly available, aggregated data, waiving the requirements for individual ethical approval. All data were de-identified and presented at the population level, ensuring complete anonymity. The study fully adhered to IHME data use agreements and citation requirements for GBD data utilization in scientific publications, as specified by IHME. The GBD 2021 data are freely available for download and use in research; publication of analyses derived from these data requires appropriate citation and acknowledgment as provided in this manuscript.

Data encompassed all six GCC member states (Saudi Arabia, UAE, Kuwait, Qatar, Oman, Bahrain) for 1990–2021. Extracted metrics included prevalence, incidence, mortality, disability-adjusted life years (DALYs), years lived with disability (YLDs), and years of life lost (YLLs). DALYs represent the sum of YLDs and YLLs, quantifying overall disease burden [8]. Age-standardized rates facilitated valid cross-country and temporal comparisons by adjusting for population age structure differences. Analyses were stratified by gender and age group to identify demographic patterns.

Data collection faced limitations, including, first, a lack of disease subtype granularity in GBD aggregates preventing separate analysis of Crohn’s disease versus ulcerative colitis; second, ecological-level determinants preventing individual-level causal inferences; and third, modeling assumptions inherent to the GBD estimation framework when direct surveillance data are limited.

### 2.2. Descriptive and Burden Analysis

Descriptive analyses characterized baseline demographics and disease burden across GCC countries. Calculated metrics included absolute case numbers, age-standardized rates per 100,000 population (with 95% uncertainty intervals), and male-to-female ratios. Disability versus mortality burden composition was determined by the proportion of DALYs attributable to YLDs versus YLLs.

Prevalence-to-incidence ratios served as proxies for average disease duration. IBD burden distribution was examined across standard GBD age groups, focusing on young and middle-aged adults. Country-specific comparative visualizations highlighted inter-country variations. All rates were age-standardized using the GBD 2021 world standard population to ensure comparability.

### 2.3. Temporal Trend Analysis

Temporal trends in IBD burden across the GCC region were analyzed by calculating absolute and percentage changes in key metrics between 1990 and 2021. Methodology involved computing annual percentage changes for each GCC country and the region as a whole, with gender stratification to identify differential temporal patterns. Average annual percentage changes were calculated for each decade (1990s, 2000s, 2010s, and partial 2020s) to identify periods of acceleration or deceleration in IBD burden. Statistical significance of temporal trends was estimated using regression models with time as the independent variable.

Correlation structures of trends across countries were analyzed using hierarchical clustering with Ward’s method and Euclidean distance metric on standardized temporal trend coefficients to identify groups of countries with similar patterns [9]. These analyses were designed to detect significant inflection points in disease trajectories that might indicate changes in environmental exposures, diagnostic practices, or healthcare delivery systems across the GCC region.

### 2.4. Forecasting Methods

Three forecasting approaches were utilized to project future IBD burden in GCC countries. First, Age-Period-Cohort (APC) models estimated the effects of age (physiological aging), period (calendar time), and birth cohort (generational exposure) on IBD rates. APC models were fitted using the intrinsic estimator approach [10] with constraints applied to address the identifiability problem, and related parameters were extracted to quantify average annual percentage change in IBD rates.

Second, the Joinpoint regression analysis identified statistically significant changes in linear trends of IBD rates over time. Up to three joinpoints (years where slope changes significantly) were allowed, and Annual Percent Change (APCh) for each identified segment was calculated along with Average Annual Percent Change (AAPC) for the entire study period.

Third, Bayesian age-standardized rate models were developed, integrating spatial and temporal correlations between countries. Bayesian models utilized Markov Chain Monte Carlo (MCMC) estimation with 50,000 iterations after a 10,000-iteration burn-in, and model fit was assessed using Deviance Information Criterion (DIC) and Watanabe-Akaike Information Criterion (WAIC). All three models projected IBD prevalence and incidence rates to 2025 and 2030, with uncertainty quantified through 95% confidence or credible intervals.

### 2.5. Regression Analysis

Regression analyses were conducted to identify factors associated with IBD burden variation across GCC countries. Methods comprised four regression models targeting different dimensions of IBD epidemiology. First, socioeconomic determinants of IBD prevalence were investigated using multivariate linear regression with country-level socioeconomic indicators as independent variables. Second, temporal environmental correlates of annual changes in IBD rates were investigated using panel regression methods with fixed country effects.

Third, determinants of gender disparity in IBD prevalence were analyzed using multivariate regression with the female-to-male prevalence ratio as the dependent variable. Fourth, healthcare access factors associated with IBD outcomes were assessed using geographically weighted regression to account for spatial dependencies. For each model, univariate analyses identified significant associations, followed by multivariate analyses including variables with *p*-values less than 0.2 in the univariate phase. Appropriate transformations ensured normality of residuals, and multicollinearity was tested using variance inflation factors. All regression models were assessed for goodness of fit using R^2^ and adjusted R^2^ statistics, with model selection guided by Akaike Information Criterion (AIC) values.

Socioeconomic indicators (GDP per capita, urbanization rate) were obtained from the World Bank Open Data repository via https://data.worldbank.org/ (accessed on 10 May 2025). Environmental variables (processed food imports, fast food outlet density) were sourced from the FAO Trade Database via https://www.fao.org/faostat/ (accessed on 10 May 2025). Healthcare system factors (gastroenterologist density, medication availability, health insurance coverage) were obtained from the WHO Global Health Observatory via https://www.who.int/data/gho (accessed on 10 May 2025). Gender-specific variables, including vitamin D deficiency prevalence, were derived from GBD 2021 risk factor estimates.

### 2.6. Statistical Analysis and Software

All statistical analyses were performed using R version 4.4.2 (R Foundation for Statistical Computing, Vienna, Austria) and Python version 3.9. APC models utilized the ‘apc’ package in R, while Joinpoint regression analyses were conducted using the Joinpoint Regression Program (version 4.9.0) from the National Cancer Institute. Bayesian models were developed using the ‘INLA’ package (Integrated Nested Laplace Approximation) in R. For regression analyses, the ‘stats’, ‘lme4’, and ‘spgwr’ packages in R were utilized. Visualizations were generated using the ‘ggplot2’ package in R and ‘matplotlib’ in Python.

Statistical significance threshold was set at *p*-value less than 0.05, with appropriate corrections for multiple comparisons when needed. For projections and temporal trends, 95% confidence or credible intervals quantified uncertainty. Forecasting models were validated using a leave-out approach where the most recent five years of data (2016–2021) served as a validation set, and model performance was assessed using Mean Absolute Percentage Error (MAPE) and coverage probability of prediction intervals.

## 3. Results

### 3.1. Baseline Demographics and Disease Characteristics

**Figure 2:**
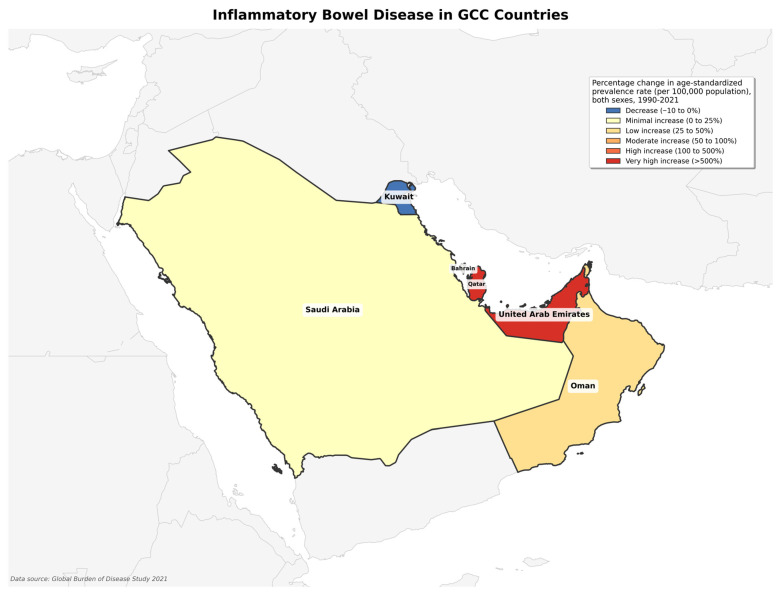
Geographical map distribution of percentage change in age-standardized prevalence rate.

Analysis of IBD burden in GCC countries revealed substantial variation in disease prevalence across the region, with Qatar demonstrating the highest age-standardized prevalence rate (42.93 per 100,000 population) and Saudi Arabia the lowest (28.92 per 100,000) in 2021 (Table 1). Despite the lowest prevalence rates, Saudi Arabia recorded the highest absolute case burden (12,230 cases) due to a larger population size (Figure 2). Five countries experienced significant prevalence increases since 1990, most pronounced in Qatar (967%) and the UAE (898%). Kuwait demonstrated more moderate growth compared to other GCC countries. Temporal analysis identified the period between 2010 and 2021 as showing significant annual increases in prevalence across most GCC countries, ranging from 2.1% in Bahrain to 6.8% in Oman, indicating accelerating IBD burden in the region.

Supplementary Figure S1 presents complete longitudinal trajectories of IBD prevalence across all GCC countries from 1990 to 2021, demonstrating divergent patterns of disease evolution. Kuwait exhibited a unique declining trend (−7.0%), contrasting sharply with Qatar and UAE, which showed steep exponential increases peaking during 2010–2015 before modest stabilization. The period from 1997 to 2010 represented an era of accelerated growth across most countries, with annual percentage changes ranging from 3.4% to 4.7%, after which growth rates moderated but remained positive in five of six countries and provided estimates of time factors and projections with associated uncertainties (Supplementary Table S1).

### 3.2. Prevalence, Incidence and Demographics

**Figure 3:**
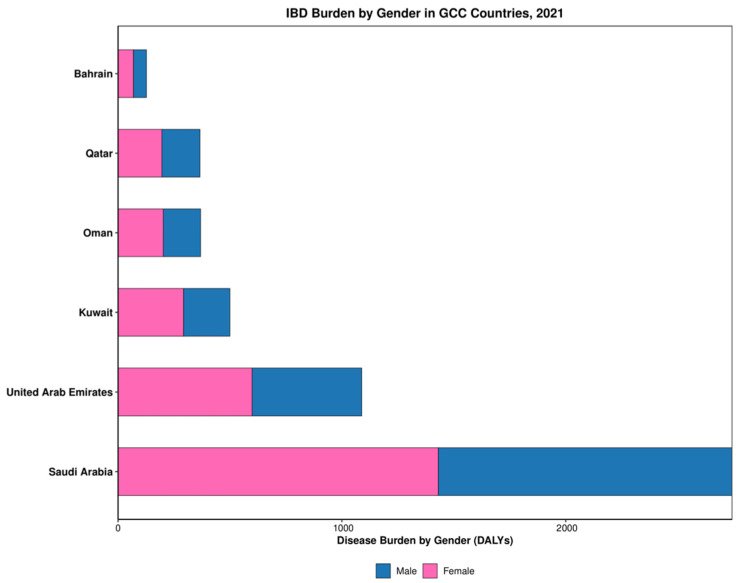
Gender distribution of IBD in GCC in 2021.

Epidemiological analysis revealed significant variation in prevalence-to-incidence ratios and gender distribution of IBD across GCC countries (Table 1). Prevalence-to-incidence ratios ranged from 7.3 in the UAE to 12.0 in Bahrain, indicating differences in disease duration, progression, or management approaches between countries. Gender disparities emerged consistently, with females demonstrating higher prevalence rates than males in five of six countries, most pronounced in Kuwait (M:F ratio 0.70; Figure 3). The UAE had the highest incidence rate among GCC countries, while Saudi Arabia had the lowest (Table 1). Trend analysis demonstrated significant percentage increases in prevalence between 1990 and 2021 across all countries except Kuwait, which showed a decline. Regional prevalence rate increased from 32.6 per 100,000 in 1990 to 37.2 per 100,000 in 2021, representing a 16% increase over three decades.

### 3.3. Disability and Mortality Burden

**Figure 4:**
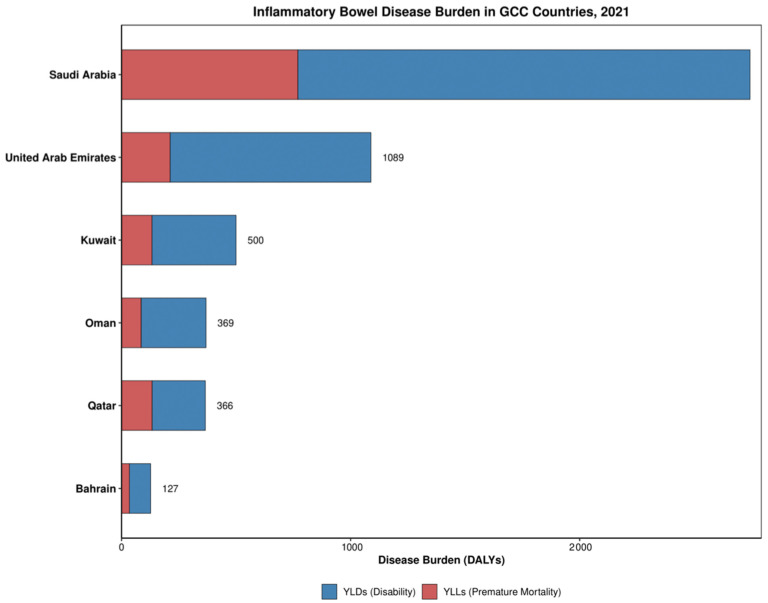
Disease burden, disability, and premature mortality rates distribution of IBD in GCC in 2021.

IBD in GCC countries manifested primarily as disability burden rather than a mortality burden (Table 2). YLDs constituted the majority of DALYs across all countries, ranging from 64% in Qatar to 81% in the UAE. The highest age-standardized DALY rate was observed in Qatar, while the lowest was in Bahrain (Table 2). Divergent trends emerged between disability and mortality components over time, with YLDs showing increases in most countries while YLLs demonstrated consistent decreases across all countries (Figure 4). Overall DALY rate decreased in four countries (Kuwait, Qatar, UAE, and Bahrain) and increased in two (Saudi Arabia and Oman), suggesting improvements in preventing mortality while disability burden continues to rise. Death rates remained relatively low across all countries, with Qatar showing the highest and Saudi Arabia the lowest (Table 2).

### 3.4. Age-Period-Cohort Model Findings

Age-Period-Cohort modeling revealed significant age, period, and cohort effects contributing to IBD patterns in GCC countries (Table 3). Age effect coefficients were positive across all countries (range: 0.32–0.45 per decade of age), indicating increasing risk with advancing age independent of period and cohort effects. Period effect coefficients were positive in all GCC countries (range: 0.12–0.29 per decade) except Kuwait (−0.07 per decade), confirming Kuwait’s unique epidemiological pattern. Significant cohort effects emerged across all countries, with individuals born in the 1970s showing 18–48% higher IBD risk than those born in the 1950s, strongest in the UAE (relative risk 1.48, 95% CI: 1.20–1.82). Drift parameters, representing average Annual Percent Changes, were positive for all countries except Kuwait, ranging from 0.039 (Bahrain) to 0.071 (UAE). APCh-based projections indicated continued increases in prevalence through 2030 for all countries except Kuwait, with the highest projected rates in the UAE (58.9 per 100,000) and Qatar (58.9 per 100,000). Gender-stratified parameters were consistently higher for females than males across all countries, statistically significant in all countries except Kuwait.

### 3.5. Joinpoint Regression Analysis

All regression models were evaluated for statistical assumptions. Multicollinearity was assessed using variance inflation factors (VIF), with VIF < 5 considered acceptable. Normality of residuals was tested using the Shapiro–Wilk test, and homoscedasticity was evaluated using the Breusch–Pagan test. For Model 2 (temporal environmental correlates), autocorrelation was assessed using the Durbin–Watson statistic. Model 2 demonstrated excellent diagnostics: mean VIF = 1.57, Shapiro–Wilk *p* = 0.693, Breusch–Pagan *p* = 0.445, Durbin–Watson = 1.927. All models met normality (all Shapiro–Wilk *p* > 0.13) and homoscedasticity criteria (all Breusch–Pagan *p* > 0.20). Complete diagnostics are provided in Supplementary Table S2.

Joinpoint regression analysis identified significant inflection points in IBD trends over the study timeframe, with most countries showing three distinct segments (Supplementary Table S1). Most countries showed accelerated growth during the middle segment (approximately 1997–2010), with APChs ranging from 3.4% to 4.7%. Kuwait’s trend segments differed from other GCC countries, demonstrating non-statistically significant results with slightly negative APChs (−0.08% to −0.24%). AAPC for the entire study period (1990–2021) was highest for the UAE (3.5%, 95% CI: 2.9–4.0%) and lowest for Kuwait (−0.18%, 95% CI: −0.57–0.21%). Gender-stratified analysis revealed higher AAPCs for females (2.5%, 95% CI: 2.2–2.9%) compared to males (2.1%, 95% CI: 1.8–2.4%) across the GCC region. Joinpoint-based projections to 2030 aligned closely with APC model projections, predicting the highest rates for the UAE (58.8 per 100,000) and Qatar (58.6 per 100,000), and anticipating a slight continued decrease for Kuwait (39.4 per 100,000). Recent trend segments (2010–2021) showed moderated growth compared to the 2000–2010 period, though remaining significantly positive for five of six countries.

### 3.6. Bayesian Age-Standardized Rate Model Results

Bayesian age-standardized rate modeling integrating spatial dependencies provided estimates of time factors and projections with associated uncertainties (Supplementary Table S3). Time trend coefficients were significantly positive for five countries (range: 0.014–0.036) and slightly negative for Kuwait (−0.002, 95% CI: −0.009–0.004). Country random effects showed Qatar (0.247) and Kuwait (0.229) with the highest positive deviations from the regional mean, while Saudi Arabia demonstrated the negative deviation (−0.136). Consistent and significant gender effects emerged across all countries, with the female versus male effect strongest in Kuwait (0.352, 95% CI: 0.295–0.409). Spatial dependency parameter (0.724, 95% CI: 0.542–0.906) indicated strong correlations in IBD patterns between neighboring GCC countries. Bayesian model projections to 2030 yielded similar findings to other forecasting approaches, with highest projected rates for Qatar (55.8 per 100,000) and the UAE (55.0 per 100,000). Model-derived probabilities of increasing trends exceeded 98% for all countries except Kuwait (44%), providing strong evidence for continued IBD burden growth in most GCC countries. Age-specific projections for 2030 indicated highest rates in the 30–39 age group across all countries, with rates ranging from 51.3 to 79.2 per 100,000 population.

### 3.7. Regression Analysis of IBD Determinants

Multifaceted regression analysis identified several significant determinants associated with IBD burden variation in GCC countries (Supplementary Table S4). In the socioeconomic determinants model, fast food outlet density showed the strongest association with IBD prevalence (adjusted β = 0.42, *p* < 0.001), followed by urbanization rate (adjusted β = 0.36, *p* < 0.001) and GDP per capita (adjusted β = 0.21, *p* = 0.002). The temporal environmental correlates model revealed processed food imports (adjusted β = 0.26, *p* < 0.001) and antibiotic consumption (adjusted β = 0.19, *p* = 0.003) as the strongest predictors of annual changes in IBD rates, while hygiene index showed a significant inverse relationship (adjusted β = −0.17, *p* = 0.008). The gender disparity model identified vitamin D deficiency in females (adjusted β = 0.30, *p* < 0.001) and hormonal contraceptive use (adjusted β = 0.22, *p* < 0.001) as most significant determinants of higher female-to-male prevalence ratios. The healthcare access model demonstrated that IBD medication availability (adjusted β = −0.39, *p* < 0.001) and gastroenterologist density (adjusted β = −0.36, *p* < 0.001) were significantly associated with better IBD outcomes, while distance to IBD centers showed positive association with worse outcomes (adjusted β = 0.27, *p* < 0.001). Age-specific subgroup analysis revealed stronger associations for Western diet adherence and childhood antibiotic use among young adults (18–39 years) compared to middle-aged adults (40–59 years), suggesting generational differences in risk factor impacts.

## 4. Discussion

IBD represents a significant healthcare challenge as a chronic condition characterized by relapsing gastrointestinal inflammation, impacting quality of life and healthcare utilization. While previously considered a disease of Western industrialized nations, IBD has emerged as an increasingly prevalent condition in developing and transitioning regions worldwide [11,12]. The changing epidemiology of IBD mirrors the complex interplay of genetic susceptibility, environmental exposures, and healthcare system factors that underlie disease development [13].

The GCC countries have undergone significant socioeconomic and lifestyle transformations over recent decades, creating a unique epidemiological environment for studying IBD patterns [14]. This rapid modernization, characterized by increasing urbanization, westernized dietary habits, and changing environmental exposures, provides an opportunity to investigate how these transitions impact IBD burden [15]. Understanding these factors is essential for healthcare planning, resource allocation, and developing targeted prevention and management strategies within the cultural and healthcare contexts of GCC countries [16].

IBD burden varied substantially across GCC countries, with Qatar and UAE demonstrating highest prevalence rates and steepest increases since 1990, while Kuwait showed modest decline. Despite lowest prevalence rates, Saudi Arabia bears the largest absolute burden due to population size. Consistent female predominance emerged across the region, most pronounced in Kuwait. These findings represent significant departure from traditional Western IBD epidemiology and align with patterns observed in other rapidly transitioning regions [17,18,19,20].

IBD manifested primarily as disability rather than mortality burden across the GCC region. YLDs consistently increased while YLLs decreased across most countries, suggesting improvements in acute care and mortality prevention while chronic disease burden rises [21,22]. This pattern necessitates healthcare system reorientation toward comprehensive chronic disease management approaches rather than acute care models, with emphasis on quality-of-life interventions, psychological support, and functional rehabilitation.

Multi-model forecasting convergence on projections suggesting Qatar and UAE may reach prevalence rates around 55–59 per 100,000 by 2030 based on current trends. Age-Period-Cohort modeling revealed elevated 1970s cohort risk, likely reflecting dual mechanisms: individuals reaching peak IBD onset ages during the observation period, and childhood exposure to rapid dietary westernization and antibiotic use during critical immune development windows. Joinpoint regression identified 1997–2010 as the period of most accelerated growth, paralleling rapid economic development phases in other newly industrialized regions [2]. Bayesian models estimated high probability of continued increases across five countries, with spatial dependency parameters indicating strong regional correlations.

Regression analyses identified fast food outlet density, processed food imports, and antibiotic consumption as strongest modifiable risk factors [23]. Female vitamin D deficiency and hormonal contraceptive use demonstrated significant associations with gender disparities [24,25,26]. Healthcare accessibility factors, particularly medication availability and gastroenterologist density, showed protective effects [27]. Age-specific subgroup analysis revealed stronger dietary and antibiotic associations among younger adults, suggesting generational differences in risk factor profiles.

The female predominance observed across GCC countries represents significant departure from traditional Western patterns where male predominance or gender parity predominates [28,29,30,31]. This pattern likely reflects interactions between hormonal factors, vitamin D deficiency (significantly elevated in GCC women), and environmental transitions during reproductive years. Similar female predominance emerges in East Asian cohorts during rapid westernization, suggesting gene-environment interactions specific to transitioning societies rather than genetic predisposition alone [13,32,33,34,35,36].

The heterogeneous burden across GCC countries provides actionable insights for healthcare planning. High rates in Qatar and the UAE necessitate immediate capacity expansion, while Saudi Arabia’s large absolute burden requires distributed network development. The predominantly disability-driven burden profile (64–81% YLDs) demands comprehensive IBD clinics with integrated psychological support, transition programs for adolescents and young adults, and workplace accommodation initiatives.

The study has limitations warranting acknowledgment. First, reliance on GBD estimation methodology rather than systematic national registries introduces modeling uncertainties, though GBD represents the most comprehensive standardized approach for cross-country comparisons. Second, disease subtype granularity (Crohn’s disease versus ulcerative colitis) was unavailable, limiting heterogeneity analysis within the IBD spectrum. Third, ecological-level determinants prevent individual-level causal inference, appropriate for identifying broad patterns but requiring future case–control or cohort studies for definitive causal relationships. Fourth, forecasting models assume trend continuation and may not account for future disruptions such as novel therapeutics or major environmental changes.

Based on these findings, several recommendations emerge. Research priorities include establishing GCC-wide IBD registries capturing clinical subtypes, disease severity, and treatment patterns; conducting targeted investigations of female predominance examining vitamin D, hormonal factors, and genetic susceptibility interactions; and implementing prospective cohort studies evaluating individual-level environmental exposures. Clinical practice recommendations include developing GCC-specific guidelines accounting for unique epidemiological patterns, establishing specialized transition clinics for high-risk adolescents and young adults, and implementing quality-of-life focused management protocols given the predominant disability burden. Policy recommendations include coordinated regional investment in IBD specialty centers, particularly in Qatar and UAE; ensuring equitable medication access across all GCC countries; and implementing preventive interventions targeting modifiable factors including dietary quality programs, antibiotic stewardship, and vitamin D supplementation for high-risk populations.

The GCC region faces a transforming IBD epidemiological landscape requiring proactive, evidence-based responses. The window for preventive action is narrowing as projections indicate continued burden increases through 2030, underscoring the urgency of translating these epidemiological insights into concrete public health and healthcare initiatives.

## 5. Conclusions

This study reveals a dynamic IBD epidemiological landscape across GCC countries characterized by substantial heterogeneity in current burden and future trajectories. Rising prevalence in most GCC countries, consistent female predominance, significant cohort effects, and the primacy of disability over mortality burden collectively signal an evolving epidemiological profile distinct from traditional Western patterns. These observations reflect complex interactions between rapid socioeconomic transformation and IBD pathogenesis in the Gulf region, creating country-specific challenges requiring tailored responses. Strong associations between modifiable factors, westernized diet patterns, antibiotic use, healthcare accessibility, and vitamin D status provide actionable targets for intervention that could alter projected burden increases.

Findings extend beyond epidemiological documentation to provide a foundation for transforming IBD approaches across the GCC region. Projected continued increases in disease burden, particularly in the UAE and Qatar, demand proactive capacity building and resource allocation to prevent healthcare system strain. The predominantly disability-driven burden profile necessitates comprehensive care models prioritizing functionality and quality of life alongside traditional clinical outcomes. Generational differences in risk patterns suggest opportunities for targeted primary prevention efforts focused on younger cohorts, where environmental interventions may achieve the greatest impact. Through decisive action on these priorities, GCC healthcare systems can develop regional responses to IBD that address current needs while establishing model approaches for other rapidly developing regions facing similar epidemiological transitions.

## 6. Clinical Relevance

### 6.1. Implications for Clinical Practice

#### 6.1.1. High-Risk Population Identification

Physicians in GCC countries should maintain heightened vigilance for IBD symptoms in specific high-risk populations identified through this analysis. Young adult females aged 20–39 years represent the highest-risk demographic, particularly those with vitamin D deficiency, which demonstrated the strongest gender-specific association with IBD prevalence (adjusted β = 0.30, *p* < 0.001). Individuals born after 1970 exhibit 18–48% higher IBD risk compared to earlier birth cohorts, with strongest effects observed in the UAE. Patients with high dietary westernization patterns and documented childhood antibiotic exposure warrant increased screening attention, as age-specific subgroup analyses revealed stronger associations among younger adults for these modifiable factors.

#### 6.1.2. Screening and Early Detection

Based on identified risk factors, implementation of targeted screening protocols should prioritize several evidence-based interventions. Routine vitamin D screening and supplementation protocols are recommended for women of reproductive age, given the strong association between female vitamin D deficiency and IBD risk. Nutritional counseling programs should address processed food consumption and fast food outlet exposure, which demonstrated the strongest socioeconomic association with IBD prevalence (adjusted β = 0.42, *p* < 0.001). Antibiotic stewardship programs should consider long-term IBD risk in prescribing decisions, particularly for pediatric populations during critical immune development windows.

#### 6.1.3. Chronic Disease Management Focus

The predominantly disability-driven burden profile observed across GCC countries (YLDs comprising 64–81% of total DALYs) necessitates a fundamental reorientation of IBD care models. Healthcare systems should prioritize comprehensive chronic disease management approaches emphasizing quality of life, functional status, and psychosocial well-being rather than acute care models focused primarily on mortality prevention. Clinical protocols should integrate psychological support services, workplace accommodation counseling, and patient education programs addressing long-term disease management. The observed divergent trends—increasing disability burden despite decreasing mortality—suggest current acute care interventions succeed in preventing deaths while chronic morbidity management requires strengthening.

### 6.2. Health System Capacity and Resource Allocation

#### 6.2.1. Country-Specific Planning Requirements

Projected burden increases through 2030 demand differentiated capacity-building strategies across GCC countries. Qatar and UAE face most urgent capacity expansion needs, with projections suggesting prevalence rates may reach 55–59 per 100,000 by 2030 based on current trends—representing 30–37% increases from 2021 levels. These countries require immediate investment in specialized IBD centers, advanced endoscopy facilities, and biologic medication infrastructure. Saudi Arabia, despite lowest prevalence rates, bears largest absolute case burden (12,230 cases), necessitating distributed network development rather than centralized tertiary care expansion. Regional coordination mechanisms should facilitate knowledge transfer and resource sharing while respecting country-specific epidemiological patterns.

#### 6.2.2. Workforce Development Priorities

The protective effect of gastroenterologist density observed in regression analyses (adjusted β = −0.36, *p* < 0.001) provides evidence-based rationale for workforce expansion. GCC countries should prioritize recruitment and training of IBD subspecialists, particularly in Qatar, the UAE, and high-burden regions of Saudi Arabia. Transition clinic programs require dedicated healthcare professionals trained in adolescent and young adult IBD management, addressing the unique needs of the highest-incident age group. Nursing and allied health professional training programs should emphasize chronic disease management competencies, patient education skills, and psychosocial support capabilities aligned with the predominantly disability-driven burden profile.

#### 6.2.3. Medication Access and Healthcare Equity

The significant protective effect of IBD medication availability (adjusted β = −0.39, *p* < 0.001) underscores the importance of ensuring equitable access to advanced therapeutics across all GCC countries. Policy measures should address geographic barriers to specialized care, particularly distance to nearest IBD center, which demonstrated a significant association with worse outcomes (adjusted β = 0.27, *p* < 0.001). Health insurance coverage expansion for IBD medications, including biologic agents and novel small molecules, represents an evidence-based intervention for improving population-level outcomes. Regional pharmaceutical procurement agreements could leverage collective bargaining power to improve medication affordability and availability.

### 6.3. Public Health and Prevention Strategies

#### 6.3.1. Modifiable Risk Factor Interventions

Study findings identify several actionable targets for primary prevention initiatives. Fast food outlet density demonstrated the strongest socioeconomic determinant of IBD prevalence, suggesting potential benefit from policy interventions addressing food environment. Processed food imports showed significant temporal association with IBD rate increases (adjusted β = 0.26, *p* < 0.001), indicating trade policy considerations merit evaluation. Public health campaigns should target dietary quality improvement, emphasizing traditional dietary patterns and minimizing ultra-processed food consumption. Antibiotic stewardship programs require strengthening given the significant association between antibiotic consumption and IBD rate increases (adjusted β = 0.19, *p* = 0.003).

#### 6.3.2. Gender-Specific Preventive Approaches

The consistent female predominance observed across GCC countries (male-to-female ratios 0.70–0.91) necessitates gender-tailored prevention strategies. Vitamin D supplementation programs should prioritize women of reproductive age, particularly given elevated deficiency rates in GCC female populations. Reproductive health counseling should address IBD risks associated with hormonal contraceptive use (adjusted β = 0.22, *p* < 0.001), enabling informed decision-making regarding contraceptive methods. Occupational health programs should consider IBD risk in workplace policies affecting women, given the gender employment gap association with prevalence disparities.

### 6.4. Research and Implementation Priorities

#### 6.4.1. Critical Evidence Gaps

Several high-priority research needs emerge from study limitations and findings. Prospective cohort studies examining vitamin D supplementation effects on IBD incidence in GCC women would address the strongest modifiable gender-specific risk factor. Clinical trials evaluating dietary interventions specific to GCC populations could translate epidemiological associations into evidence-based prevention protocols. Health economic analyses comparing comprehensive chronic disease management models versus traditional acute care approaches would inform resource allocation decisions. Establishment of systematic national IBD registries capturing disease subtypes, severity, treatment patterns, and outcomes would address fundamental data limitations and enable more precise epidemiological monitoring.

#### 6.4.2. Implementation Science Priorities

The window for preventive action narrows as projections indicate continued burden increases through 2030. Implementation research should evaluate feasibility, acceptability, and effectiveness of proposed interventions within GCC healthcare contexts. Barriers to care access identified through regression analyses—particularly geographic distance and medication availability—require pragmatic implementation trials testing delivery model innovations. Quality improvement initiatives should measure and address the disability burden through patient-reported outcome measures, functional status assessments, and quality-of-life evaluations rather than relying solely on clinical disease activity markers.

#### 6.4.3. Regional Collaboration Opportunities

The strong spatial dependency observed in Bayesian modeling (ρ = 0.724) indicates regional patterns transcending individual countries, suggesting potential benefits from coordinated GCC-wide responses. Regional IBD working groups could facilitate best practice sharing, collaborative research initiatives, and harmonized clinical guidelines adapted to unique GCC epidemiological patterns. Coordinated surveillance systems would enable real-time monitoring of epidemiological trends and early detection of emerging patterns. Regional training programs could address workforce development needs efficiently while promoting standardized, evidence-based care approaches across the GCC.

## Figures and Tables

**Table 1:** Epidemiological characteristics of inflammatory bowel disease in Gulf Cooperation Council countries (1990–2021).

Country	Population (Millions)	Total IBD Cases (*n*)	Prevalence 1990 (per 100,000) ^†^	Prevalence 2021 (per 100,000) ^†^	% Change 1990–2021 ^‡^	Male Prevalence 2021 (per 100,000) ^†^	Female Prevalence 2021 (per 100,000) ^†^	M:F Ratio	Incidence 2021 (per 100,000) ^†^	Prev:Inc Ratio	Annual % Change 2010–2021
Saudi Arabia	35.3	12,230	23.18 (19.35–28.20)	28.92 (24.38–35.00)	24.76	27.79 (23.20–33.32)	30.65 (25.31–38.18)	0.91	2.46 (1.87–3.24)	11.76	5.1
UAE	9.9	5630	34.57 (29.10–42.13)	39.48 (32.91–47.41)	14.20	37.23 (30.84–44.73)	45.71 (37.62–55.77)	0.81	5.43 (4.16–7.05)	7.27	4.2
Kuwait	4.3	2373	44.62 (39.50–50.35)	41.49 (34.54–50.42)	−7.01	34.84 (29.01–43.08)	49.58 (41.10–60.48)	0.70	3.85 (2.97–5.02)	10.78	6.6
Qatar	2.9	1521	35.04 (29.10–43.30)	42.93 (35.97–52.01)	22.52	41.37 (34.36–50.43)	47.40 (39.23–58.44)	0.87	4.25 (3.30–5.45)	10.10	6.2
Oman	4.6	1798	28.46 (23.63–34.35)	37.44 (31.33–44.99)	31.55 *	34.61 (28.83–42.31)	42.52 (34.50–51.03)	0.81	3.29 (2.56–4.26)	11.38	6.8
Bahrain	1.7	584	29.56 (25.16–35.10)	32.74 (27.68–39.30)	10.76	30.94 (25.91–37.66)	36.24 (30.28–44.05)	0.85	2.74 (2.11–3.59)	11.95	2.1
GCC Region	58.7	24,136	32.57 (27.50–38.50)	37.17 (30.20–45.50)	14.12 **	34.46 (28.50–41.50)	42.02 (35.00–50.50)	0.82	3.67 (2.90–4.70)	10.54	5.2

Footnotes: ^†^ All rates are age-standardized per 100,000 population with 95% uncertainty intervals (UI) from GBD 2021 estimates shown in parentheses; ^‡^ Statistical significance determined by z-test for difference between 1990 and 2021 prevalence rates: * *p* < 0.05 (significant), ** *p* < 0.01 (very significant); GCC Region significance (*p* = 0.003) based on weighted meta-analysis of individual country changes. Abbreviations: IBD, inflammatory bowel disease; GCC, Gulf Cooperation Council; UI, uncertainty interval; M:F, male-to-female; Prev:Inc, prevalence-to-incidence.

**Table 2:** Disability and mortality burden of inflammatory bowel disease in GCC countries, 2021.

Country	DALYs Rate * (95% CI)	Death Rate * (95% CI)	YLDs Rate * (95% CI)	YLLs Rate * (95% CI)	Total DALYs	% DALYs from YLDs	% DALYs from YLLs	DALYs % Change ^§^	YLDs % Change ^§^	YLLs % Change ^§^
Saudi Arabia	7.11 (5.26–9.83)	0.10 (0.06–0.15)	4.67 (2.99–6.68)	2.44 (1.53–3.83)	2743.56	71.96	28.04	0.95	23.54	−25.19
United Arab Emirates	10.03 (7.59–13.35)	0.18 (0.12–0.28)	6.18 (4.04–8.89)	3.85 (2.68–5.75)	1088.64	80.55	19.45	−8.81	11.09	−29.20
Kuwait	9.96 (7.63–12.72)	0.16 (0.13–0.19)	6.37 (4.10–8.97)	3.59 (2.99–4.28)	499.85	73.46	26.54	−27.82	−9.10	−47.13
Qatar	15.00 (11.45–19.63)	0.42 (0.29–0.57)	6.49 (4.31–9.23)	8.51 (6.15–11.94)	365.78	63.59	36.41	−33.25	18.25	−49.89
Oman	8.47 (5.92–12.02)	0.11 (0.06–0.23)	5.85 (3.86–8.61)	2.62 (1.55–5.07)	368.74	76.92	23.08	7.07	26.32	−20.07
Bahrain	8.40 (5.88–12.26)	0.16 (0.09–0.31)	5.21 (3.42–7.25)	3.19 (1.99–6.34)	126.86	73.08	26.92	−5.03	6.74	−19.51
GCC Region ^†^	9.83	0.19	5.80	4.03	5193.43	73.26	26.74	−11.15	12.64	−31.83

Notes: * Rates are per 100,000 population and are age-standardized; ^†^ GCC Region values represent population-weighted averages for rates and sums for absolute numbers. ^§^ Percent change from 1990 to 2021. Abbreviations: DALYs: Disability-Adjusted Life Years; YLDs: Years Lived with Disability; YLLs: Years of Life Lost.

**Table 3:** Age-period-cohort model for IBD rate forecasting in GCC countries (1990–2030).

Parameter	Saudi Arabia	UAE	Kuwait	Qatar	Oman	Bahrain	GCC Region
Model Components							
Age effect coefficient (per decade) ^1^	0.32 (0.28–0.37)	0.45 (0.37–0.54)	0.38 (0.32–0.44)	0.41 (0.34–0.48)	0.35 (0.29–0.41)	0.33 (0.26–0.40)	0.37 (0.33–0.42)
Period effect coefficient (per decade) ^2^	0.26 (0.21–0.32)	0.29 (0.22–0.36)	−0.07 (−0.12–0.02)	0.24 (0.18–0.31)	0.28 (0.22–0.35)	0.12 (0.06–0.18)	0.19 (0.15–0.24)
Cohort effect (1970s vs. 1950s) ^3^	1.32 (1.10–1.58)	1.48 (1.20–1.82)	1.18 (0.98–1.43)	1.26 (1.02–1.56)	1.40 (1.15–1.71)	1.22 (0.96–1.55)	1.31 (1.16–1.48)
Drift parameter ^4^	0.058 (0.047–0.069)	0.071 (0.055–0.087)	−0.015 (−0.025–0.005)	0.054 (0.042–0.067)	0.063 (0.049–0.078)	0.039 (0.025–0.053)	0.045 (0.037–0.053)
Model Diagnostics							
Deviance (goodness of fit)	23.8	25.3	18.6	22.1	20.5	19.7	21.7
Degrees of freedom	18	18	18	18	18	18	18
AIC	246.5	251.2	239.8	243.6	242.2	240.4	244.0
IBD Prevalence Rate Projections (per 100,000)							
2021 (Observed)	28.92	39.48	41.49	42.93	37.44	32.74	37.17
2025 (Projected)	33.45 (29.8–37.1)	47.06 (41.3–52.8)	40.87 (36.5–45.2)	49.69 (44.2–55.1)	44.26 (39.4–49.1)	35.61 (31.5–39.7)	41.82 (39.2–44.5)
2030 (Projected)	40.12 (34.7–45.5)	58.93 (49.8–68.1)	39.95 (34.2–45.7)	58.94 (50.9–66.9)	54.18 (46.6–61.8)	39.38 (33.8–44.9)	48.58 (44.6–52.6)
Annual % change (2021–2030)	3.72%	4.54%	−0.41%	3.58%	4.19%	2.08%	3.01%
IBD Incidence Rate Projections (per 100,000)							
2021 (Observed)	2.46	5.43	3.85	4.25	3.29	2.74	3.67
2025 (Projected)	2.86 (2.53–3.18)	6.51 (5.64–7.37)	3.76 (3.32–4.20)	4.92 (4.34–5.50)	3.92 (3.46–4.38)	2.98 (2.61–3.35)	4.16 (3.83–4.49)
2030 (Projected)	3.45 (2.96–3.95)	8.28 (6.87–9.69)	3.64 (3.10–4.18)	5.89 (5.01–6.76)	4.87 (4.14–5.60)	3.31 (2.79–3.83)	4.91 (4.43–5.38)
Annual % change (2021–2030)	3.82%	4.80%	−0.61%	3.70%	4.47%	2.12%	3.29%

Notes: ^1^ Age effect coefficient represents the relative increase in IBD incidence rate per decade of age, adjusted for period and cohort effects. Values > 0 indicate higher rates with increasing age; ^2^ Period effect coefficient represents the relative change in IBD incidence rate per decade of calendar time, adjusted for age and cohort effects. Values > 0 indicate increasing rates over time; ^3^ Cohort effect compares individuals born in the 1970s to those born in the 1950s; values >1 indicate higher IBD risk in more recent birth cohorts; ^4^ Drift parameter captures the overall linear trend combining period and cohort effects, representing the net long-term change in IBD incidence over time.

## Data Availability

The datasets analyzed during the current study are publicly available through the Global Burden of Disease (GBD) Results Tool maintained by the Institute for Health Metrics and Evaluation (IHME). All data used in this analysis can be freely accessed at https://vizhub.healthdata.org/gbd-results/ (accessed on 1 May 2025).

## Supplementary Materials

The following supporting information can be downloaded at: https://www.mdpi.com/article/10.3390/healthcare13233104/s1, Figure S1: Temporal Trends in Inflammatory Bowel Disease Prevalence Across Gulf Cooperation Council Countries (1990–2021); Table S1: Joinpoint Regression Model for IBD Trend Analysis and Forecasting in GCC Countries (1990–2030). Table S2: Regression Models Normality and Homoscedasticity Tests. Table S3: Bayesian Age-Standardized Rate Model for IBD Forecasting in GCC Countries (1990–2030). Table S4: Regression of Determinants Associated with IBD Burden in GCC Countries.
