# Supplementary material for: The Burden of Inflammatory Bowel Disease in Gulf Cooperation Council Countries from 1990 to 2021 with Forecasting Projections to 2030: A Global Burden of Disease Study
Source: Healthcare (Basel). 2025 Nov 28;13(23):3104. doi: 10.3390/healthcare13233104 (PMC12691746; doi:10.3390/healthcare13233104)
Supplement: Supplementary file 1 [file healthcare-13-03104-s001.zip › healthcare-3939525-supplementary.pdf]

Supplementary Table S1: Joinpoint Regression Model for IBD Trend Analysis and Forecasting in GCC Countries (1990-2030).

Country	Time Segment	Number of Joinpoints	APC	95% CI	P-value	AAPC (1990-2021)	Observed Rate 2021	Projected Rate 2030
Saudi Arabia	1990-1999	-	1.37	(0.92-1.82)	<0.001	2.68 (2.21-3.15)	28.92	39.57 (35.76-43.38)
	1999-2009	1	3.85	(3.41-4.29)	<0.001			
	2009-2021	1	2.58	(2.26-2.90)	<0.001			
United Arab Emirates	1990-2000	-	0.84	(0.39-1.29)	<0.001	3.45 (2.86-4.04)	39.48	58.83 (52.47-65.19)
	2000-2010	1	4.72	(4.05-5.39)	<0.001			
	2010-2021	1	4.26	(3.74-4.78)	<0.001			
Kuwait	1990-2002	-	-0.08	(-0.37-0.21)	0.586	-0.18 (-0.57-0.21)	41.49	39.38 (37.81-40.95)
	2002-2012	1	-0.24	(-0.63-0.15)	0.225			
	2012-2021	1	-0.19	(-0.53-0.15)	0.274			
Qatar	1990-1997	-	0.56	(0.11-1.01)	0.015	2.64 (2.08-3.20)	42.93	58.61 (52.83-64.39)
	1997-2007	1	3.42	(2.86-3.98)	<0.001			
	2007-2021	1	3.24	(2.84-3.64)	<0.001			
Oman	1990-1998	-	1.28	(0.76-1.80)	<0.001	2.84 (2.37-3.31)	37.44	52.61 (47.58-57.64)
	1998-2010	1	3.74	(3.31-4.17)	<0.001			
	2010-2021	1	3.08	(2.69-3.47)	<0.001			
Bahrain	1990-1995	-	0.52	(-0.12-1.16)	0.109	1.25 (0.84-1.66)	32.74	38.37 (36.11-40.63)
	1995-2007	1	1.87	(1.48-2.26)	<0.001			
	2007-2021	1	1.07	(0.81-1.33)	<0.001			
GCC Region	1990-1997	-	0.98	(0.69-1.27)	<0.001	2.31 (2.01-2.61)	37.17	49.58 (46.42-52.74)
	1997-2008	1	3.12	(2.82-3.42)	<0.001			

Supplementary Table S2: Regression Models Normality and Homoscedasticity Tests.

**Model**	**Mean VIF**	**Max VIF**	**Shapiro-Wilk P**	**Breusch-Pagan P**	**Durbin-Watson**
Socioeconomic	21.13	35.39	0.655	0.200	N/A†
Temporal Environmental	1.57	1.99	0.693	0.445	1.927
Gender Disparity	93.02	132.10	0.183	0.213	N/A†
Healthcare Access	260.82	627.51	0.133	0.714	N/A†

Note: † Cross-sectional models; autocorrelation testing not applicable.

Supplementary Table S3: Bayesian Age-Standardized Rate Model for IBD Forecasting in GCC Countries (1990-2030).

Parameter	Saudi Arabia	UAE	Kuwait	Qatar	Oman	Bahrain	GCC Region
Model Specifications							
Time trend coefficient (β)	0.029 (0.022-0.037)	0.036 (0.026-0.046)	-0.002 (-0.009-0.004)	0.028 (0.020-0.036)	0.031 (0.022-0.039)	0.014 (0.008-0.021)	0.023 (0.019-0.028)
Country random effect (u)	-0.136 (-0.214-0.058)	0.198 (0.112-0.285)	0.229 (0.142-0.315)	0.247 (0.158-0.335)	0.142 (0.066-0.218)	0.013 (-0.071-0.097)	—
Gender effect (female vs. male)	0.118 (0.081-0.155)	0.208 (0.158-0.258)	0.352 (0.295-0.409)	0.138 (0.085-0.191)	0.206 (0.154-0.258)	0.159 (0.101-0.217)	0.197 (0.175-0.219)
Spatial dependency (ρ)	0.724 (0.542-0.906)	Same for all countries	Same for all countries	Same for all countries	Same for all countries	Same for all countries	—
Model Diagnostics							
sDIC	189.6	193.2	187.5	190.7	188.4	186.3	1135.7 (total)
WAIC	192.1	196.5	190.3	194.2	191.9	189.7	1154.7 (total)
Effective parameters (pD)	14.3	14.6	13.8	14.5	14.1	13.6	85.9 (total)
IBD Prevalence Rate Projections (per 100,000)							
2021 (Observed)	28.92	39.48	41.49	42.93	37.44	32.74	37.17
2025 (Projected)	32.34 (28.71-36.37)	45.62 (39.84-52.14)	41.15 (36.98-45.74)	48.32 (42.67-54.68)	42.85 (37.62-48.76)	34.59 (30.82-38.79)	40.81 (37.95-43.86)
2030 (Projected)	37.26 (31.57-43.97)	55.03 (45.19-67.01)	40.74 (35.02-47.39)	55.81 (46.16-67.44)	49.95 (41.47-60.16)	37.05 (31.42-43.69)	46.31 (41.47-51.68)
Probability of increasing trend	0.998	0.999	0.442	0.999	0.999	0.982	0.995
IBD Incidence Rate Projections (per 100,000)							
2021 (Observed)	2.46	5.43	3.85	4.25	3.29	2.74	3.67
2025 (Projected)	2.74 (2.38-3.16)	6.23 (5.29-7.34)	3.81 (3.36-4.32)	4.76 (4.08-5.55)	3.74 (3.22-4.35)	2.89 (2.51-3.33)	4.03 (3.67-4.43)
2030 (Projected)	3.14 (2.56-3.85)	7.45 (5.88-9.45)	3.77 (3.16-4.49)	5.47 (4.37-6.84)	4.36 (3.51-5.42)	3.11 (2.56-3.77)	4.55 (3.98-5.19)
Probability of increasing trend[7]	0.997	0.998	0.459	0.998	0.998	0.977	0.992
Forecasting Performance							
Coverage of 95% CI (2016-2021)	95.3%	94.7%	96.1%	94.9%	95.5%	95.8%	95.4%
MAPE (2016-2021)	3.26%	3.85%	2.79%	3.62%	3.47%	2.95%	3.32%
Relative bias	-0.92%	1.27%	-0.65%	1.04%	0.88%	-0.72%	0.15%
Key age-specific projections for 2030							
20-29 years	41.86 (34.39-50.98)	59.65 (47.83-74.38)	43.39 (36.61-51.36)	62.44 (50.30-77.46)	55.28 (44.79-68.23)	40.28 (33.42-48.55)	50.48 (44.16-57.67)
30-39 years	53.41 (44.28-64.34)	76.74 (62.06-94.89)	55.23 (47.01-64.83)	79.18 (64.40-97.38)	70.25 (57.54-85.74)	51.27 (42.85-61.32)	64.35 (57.02-72.65)

Supplementary Table S4: Regression of Determinants Associated with IBD Burden in GCC Countries.

**Determinant Factor**	**Univariate Analysis**			**Multivariate Analysis**		
Model 1: Socioeconomic Determinants of IBD Prevalence Rates	**β Coefficient**	**95% CI**	**P-value**	**Adjusted β**	**95% CI**	**P-value**
GDP per capita (per $1,000 increase)	0.32	(0.18-0.46)	<0.001	0.21	(0.09-0.33)	0.002
Urbanization rate (per 10% increase)	0.49	(0.31-0.67)	<0.001	0.36	(0.19-0.53)	<0.001
Fast food outlet density (per unit increase)	0.57	(0.38-0.76)	<0.001	0.42	(0.27-0.57)	<0.001
Healthcare expenditure (% of GDP)	0.18	(-0.05-0.41)	0.124	0.11	(-0.08-0.30)	0.245
Model statistics: R^2 = 0.74, adjusted R^2 = 0.71, F = 28.6, P-value<0.001						
Model 2: Temporal Environmental Correlates of Annual Change in IBD Rates						
Annual change in antibiotic consumption (DDD/1000)	0.28	(0.14-0.42)	<0.001	0.19	(0.07-0.31)	0.003
Annual change in processed food imports (%)	0.34	(0.21-0.47)	<0.001	0.26	(0.15-0.37)	<0.001
Annual change in PM2.5 levels (μg/m³)	0.14	(0.02-0.26)	0.026	0.08	(-0.03-0.19)	0.142
Annual change in hygiene index	-0.22	(-0.36-0.08)	0.003	-0.17	(-0.29-0.05)	0.008
Model statistics: R^2 = 0.68, adjusted R^2 = 0.65, F = 21.3, P-value<0.001						

Model 3: Determinants of Gender Disparity in IBD Prevalence						
Hormonal contraceptive use (per 10% increase)	0.26	(0.12-0.40)	<0.001	0.22	(0.10-0.34)	<0.001
Female-to-male smoking ratio	-0.31	(-0.47-0.15)	<0.001	-0.24	(-0.38-0.10)	0.002
Gender employment gap (per 10% decrease)	0.19	(0.04-0.34)	0.016	0.13	(0.01-0.25)	0.042
Female vitamin D deficiency prevalence (%)	0.38	(0.25-0.51)	<0.001	0.30	(0.18-0.42)	<0.001
Model statistics: $R^2 = 0.71$, adjusted $R^2 = 0.68$, $F = 24.5$, P-value<0.001						
Model 4: Healthcare Access Determinants of IBD Outcomes						
Gastroenterologist density (per 100,000)	-0.41	(-0.56-0.26)	<0.001	-0.36	(-0.49-0.23)	<0.001
Distance to nearest IBD center (per 10 km)	0.33	(0.18-0.48)	<0.001	0.27	(0.14-0.40)	<0.001
Health insurance coverage (per 10% increase)	-0.29	(-0.45-0.13)	<0.001	-0.23	(-0.37-0.09)	0.002
IBD medication availability index[3]	-0.47	(-0.63-0.31)	<0.001	-0.39	(-0.53-0.25)	<0.001
Model statistics: $R^2 = 0.79$, adjusted $R^2 = 0.76$, $F = 32.7$, P-value<0.001						
Subgroup Analysis: Age-Specific Associations with IBD Prevalence						
Young adults (18-39 years): Western diet adherence score	0.51	(0.36-0.66)	<0.001	0.43	(0.29-0.57)	<0.001
