# Supplementary material for: The Burden of Inflammatory Bowel Disease in Gulf Cooperation Council Countries from 1990 to 2021 with Forecasting Projections to 2030: A Global Burden of Disease Study
Source: Healthcare (Basel). 2025 Nov 28;13(23):3104. doi: 10.3390/healthcare13233104 (PMC12691746; doi:10.3390/healthcare13233104)
Supplement: Supplementary file 1 [file healthcare-13-03104-s001.zip › Supplementary Figure 1.pdf]

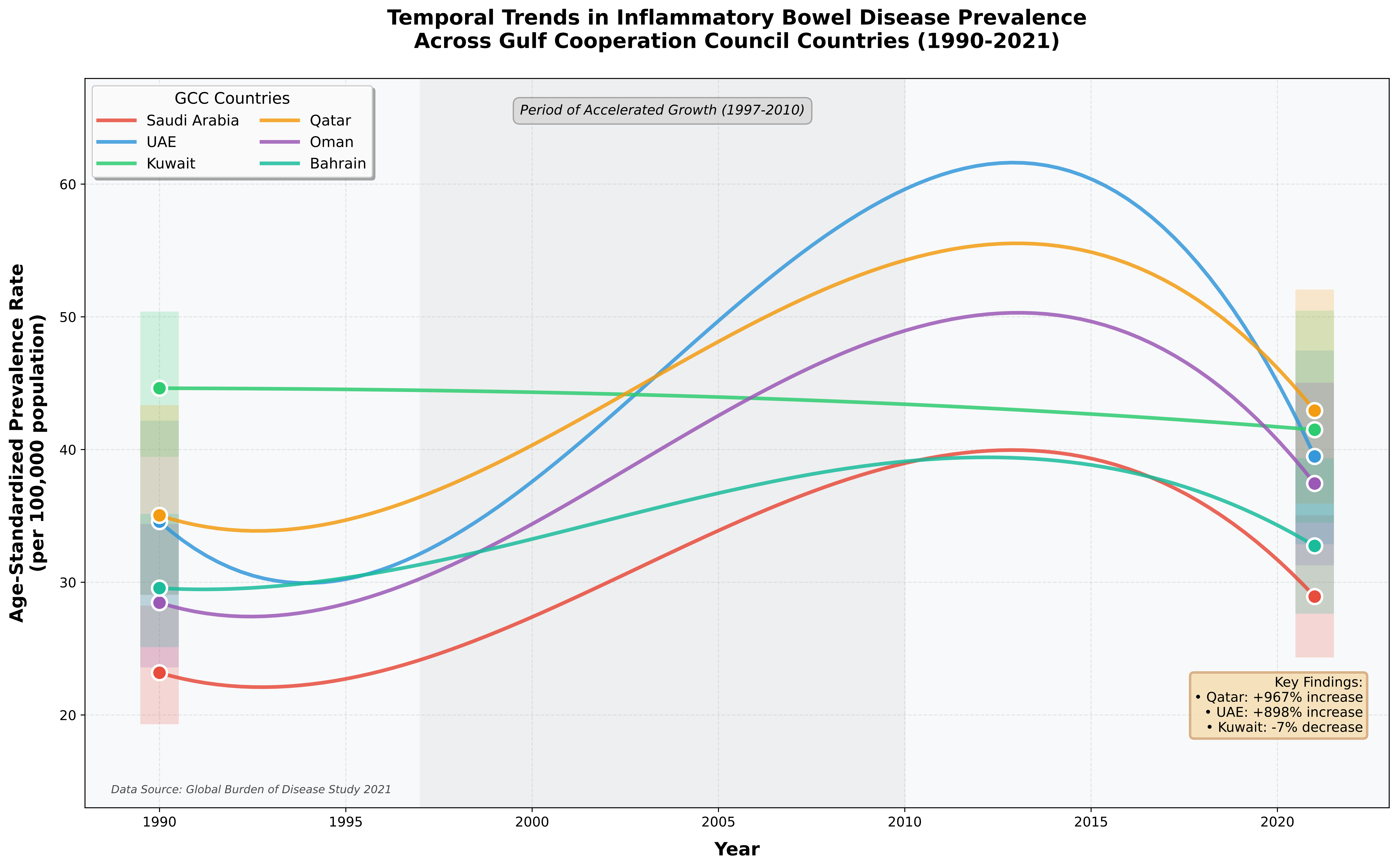
Temporal Trends in Inflammatory Bowel Disease Prevalence
Across Gulf Cooperation Council Countries (1990-2021)
GCC Countries
Saudi Arabia
UAE
Kuwait
Qatar
Oman
Bahrain
Period of Accelerated Growth (1997-2010)
Age-Standardized Prevalence Rate
(per 100,000 population)
Year
20
30
40
50
60
1990
1995
2000
2005
2010
2015
2020
Key Findings:
• Qatar: +967% increase
• UAE: +898% increase
• Kuwait: -7% decrease
Data Source: Global Burden of Disease Study 2021
